# A multicenter study on quality of life of the “greater patient” in congenital ichthyoses

**DOI:** 10.1186/s13023-021-02085-9

**Published:** 2021-10-20

**Authors:** Damiano Abeni, Roberta Rotunno, Andrea Diociaiuti, Simona Giancristoforo, Domenico Bonamonte, Carmelo Schepis, Iria Neri, Daniele Castiglia, Giovanna Zambruno, May El Hachem

**Affiliations:** 1 IDI-IRCCS, Via Monti di Creta, 104, 00167 Rome, Italy; 2 Dermatology Unit and Genodermatosis Unit, Genetics and Rare Diseases Research Division, Bambino Gesù Children’s Hospital, IRCCS, Piazza Sant’Onofrio, 4, 00165 Rome, Italy; 3 Dermatology Unit, Department of Biomedical Science and Human Oncology, University of Bari, Bari, Italy; 4 Genodermatosis Center, Oasi Scientific Institute, IRCCS, Troina, Italy; 5 Dermatology-IRCCS Policlinico di S. Orsola, Department of Experimental, Diagnostic and Specialty Medicine (DIMES), Alma Mater Studiorum University of Bologna, Bologna, Italy; 6 Genodermatosis Unit, Genetics and Rare Diseases Research Division, Bambino Gesù Children’s Hospital, IRCCS, Piazza Sant’Onofrio 4, 00165 Rome, Italy

**Keywords:** Rare skin disease, Genodermatosis, Autosomal recessive congenital ichthyosis, Lamellar ichthyosis, Congenital ichthyosiform erythroderma, Quality of life, Caregiver, Family dermatology life quality lndex, Family burden of ichthyosis

## Abstract

**Background:**

Autosomal recessive congenital ichthyoses (ARCI) are a genetically heterogeneous group of rare and chronic disorders characterized by generalized skin scaling and hyperkeratosis, erythroderma, and palmoplantar keratoderma. Additional features include ectropion, eclabium, ear deformities, foul-smell, joints contractures and walking problems, recurrent infections, as well as pruritus and pain. No curative therapy is available and disease care mainly relies on daily application of topical emollients and keratolytics to the whole-body surface. Altogether, disease signs and symptoms and treatment modalities have a major impact on quality of life of patients and their caregivers. However, very few studies have evaluated the family disease burden in ARCI.

**Methods:**

We have performed an Italian multicenter cross-sectional study to assess the secondary disease impact on family members of pediatric and adult patients with ARCI, using a validated dermatology-specific questionnaire, the family dermatology life quality index (FDLQI). Disease severity was assessed by the dermatologist in each center.

**Results:**

Seventy-eight out of 82 patients who were accompanied by at least one family member filled the FDLQI. Forty-eight (61.5%) patients were aged less than 18 years. The mean FDLQI score was 10.3 (median 10), and the most affected dimensions were (1) time needed for care, (2) extra-housework, and (3) household expenditure. Higher total FDLQI score significantly correlated with more severe disease score (*P* = 0.003). Features associated with greater family burden included recurrent infections (*P* = 0.004), foul-smell (*P* = 0.009), palmoplantar keratoderma (*P* = 0.041), but also presence of scales on the face (*P* = 0.039) and ear deformities (*P* = 0.016).

**Conclusions:**

Our findings highlight the major socio-economic and psychological burden imposed by ARCI on the QoL of family caregivers. In addition, they show that global evaluation of disease impact also on family members is an essential part of patient-reported outcomes. Finally, our data underline the need to develop specific measures for family support.

## Background

**Fig. 1:**
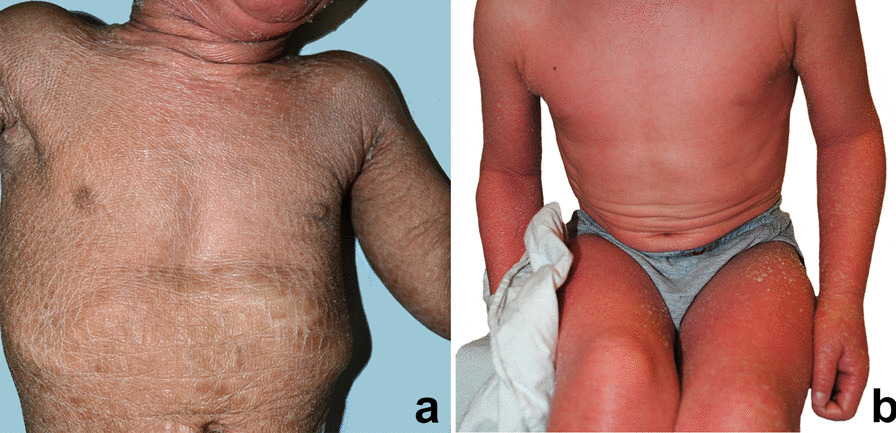
A 2-year-old female with lamellar ichthyosis due to *TGM1* mutation shows typical diffuse thick brownish adherent scales (**a**); a 3-year-old male with congenital ichthyosiform erythroderma due to *ABCA12* mutation presents generalized erythroderma with fine whitish scales (**b**)

Autosomal recessive congenital ichthyoses (ARCI) represent a genetically heterogeneous group of cornification disorders associated with mutations in at least 13 genes (*TGM1*, *ALOX12B*, *ALOXE3*, *ABCA12*, *NIPAL4*, *CYP4F22*, *PNPLA1*, *CERS3*, *SULT2B1*, *SDR9C7*, *LIPN*, *CASP14*, and *SLC27A4*) and an overall estimated prevalence of 16.2 cases per million inhabitants [1–4]. ARCI include two major clinical subtypes: lamellar ichthyosis (LI) and congenital ichthyosiform erythroderma (CIE). Disease features are severe and highly disabling. At birth the profound alteration of skin barrier, due to a collodion baby or ichthyosiform erythroderma presentation, usually requires hospitalization in neonatal intensive care unit [5]. Over time, the patients develop whole body skin scaling. LI patients present generalized thick, large, dark scales, while CIE is typified by small, thin, whitish desquamation on erythrodermic skin (Fig. 1). Palmoplantar keratoderma (PPK) is common in both LI and CIE. Additional ARCI clinical features and symptoms comprise hypohidrosis with heat intolerance, foul-smell, recurrent infections, pain, and pruritus [1, 3, 5]. Disease complications can cause functional damage including: (1) visual impairment due to ectropion and recurrent keratitis, (2) hearing defects related to scaling in the external auditory canal, and (3) walking problems consequent to joint contractures of the limbs. Moreover, skin and extracutaneous manifestations profoundly alter patients’ body image and self-perception. Due to the chronic life-long nature of the disease, patients and caregivers are confronted daily with demanding tasks. These include applying to the whole-body surface various topical treatments, specifically emollients, keratolytics, and retinoids, that may be combined with oral retinoids in more severe cases [3, 5]. Care modalities are merely symptomatic, as at present there is no curative treatment for this disease group.

ARCI manifestations and complications, together with the constant need for time-consuming care, and the relevant financial burden deriving from direct and indirect costs of ichthyosis management, profoundly impact quality of life (QoL), both of patients and their families [3, 6]. Thus, evaluation of the disease impact on family members, who are usually involved in care giving, should be part of an integrated care approach to congenital ichthyoses. However, only a single study has specifically assessed family disease burden in a small group of children suffering from different types of congenital ichthyoses [7]. More recently, a disease-specific tool to evaluate the impact of ichthyoses on family members, the Family Burden of Ichthyosis (FBI), has been developed and validated on ARCI caregivers in France [8]. An Italian version of the FBI is available [9].

We have performed an Italian multicenter cross-sectional study to assess the secondary disease impact on “the greater patient” [10], i.e., on the family members of pediatric and adult patients affected with ARCI, using the family dermatology life quality lndex (FDLQI) [11], and compared the findings with those obtained using the FBI [12].

## Results

Of 102 consecutive pediatric and adult patients seen in the involved centers, 8 refused to participate; 82 of the 94 consenting patients were accompanied by at least one family member. Of these, 78 (95.1%) (67 parents, 8 spouses, and 3 other relatives) filled the FDLQI questionnaire. Among these patients, 48 were aged less than 18 years (61.5%), and the remaining ones were adults (Table 1). Sixty-eight out of 78 patients (87.2%) were affected with LI, and 10 had CIE (12.8%). Thirty-three patients (42.3%) underwent one consultation/year, 27 (34.68%) twice a year, and 18 (23.1%) ≥ 4 consultations/year. In addition, 56 caregivers (76.7%) reported loss of working days related to the patient disease and needs. Among these 78 patients the more frequent disease signs and complications were: palmoplantar keratoderma (N = 57, 73.1%), scales on face (N = 56, 71.8%), dark scales (N = 38, 48.7%), large scales (N = 37, 47.4%), followed by foul-smell (N = 28, 35.9%), ectropion (N = 25, 32.1%), fissures (N = 25, 32.1%), recurrent infections (defined as ≥ 3 episodes/year) (N = 18, 23.1%), and external ear deformities (N = 16, 20.5%). Rare complications were eclabium (N = 5, 6.4%) and walking problems (N = 12, 15.4%). In addition, most patients complained itching (N = 69, 88.5%). Molecular diagnosis was available in 45 patients (57.7%).

**Table 1:** Association of patient sociodemographic and clinical features with the family dermatology life quality index (FDLQI) scores

Variable	Level	N	%	Mean FDLQI score	Median FDLQI score	*P* value*
Overall		78	100	10.3	10.0	
Sex	Male	35	44.9	11.0	10.0	
	Female	43	55.1	9.8	10.0	0.698
Age (years)	< 18	48	61.5	10.0	10.0	
	≥ 18	30	38.5	10.8	10.5	0.837
Clinical type	LI^a^	68	87.2	10.2	10.0	
	CIE^b^	10	12.8	8.9	9.5	0.482
Mutated gene	Undetermined	33	42.3	9.5	9.0	
	ABCA12	6	7.7	13.0	12.0	
	ALOX12B	10	12.8	11.7	12.0	
	ALOXE3	2	2.6	6.5	6.5	
	CYP4F22	5	6.4	7.6	8.0	
	NIPAL4	4	5.1	6.2	7.5	
	SDR9C7	0	0	NA	NA	
	TGM1	18	23.1	10.3	10.0	0.163
Clinical severity score^d^	Mild-moderate	56	71.8	9.2	8.0	
	Severe	22	28.2	13.1	13.0	**0.003**
Ear deformity	NO	62	79.5	9.7	8.5	
	YES	16	20.5	12.8	13.0	**0.016**
Ectropion	NO	53	67.9	9.9	9.0	
	YES	25	32.1	11.3	12.0	0.128
Eclabium	NO	73	93.6	10.1	9.0	
	YES	5	6.4	14.0	13.0	0.069
Thick scales	NO	41	52.6	9.9	9.0	
	YES	37	47.4	10.9	12.0	0.336
Dark scales	NO	40	51.3	9.1	9.5	
	YES	38	48.7	11.6	12.0	0.094
Face scales	NO	22	28.2	8.5	7.5	
	YES	56	71.8	11.0	11.0	**0.039**
Fissures	NO	53	67.9	9.8	9.0	
	YES	25	32.1	11.4	11.0	0.209
PPK^c^	NO	21	26.9	8.6	6.0	
	YES	57	73.1	11.0	11.0	**0.041**
Itch	NO	9	11.5	9.6	11.0	
	YES	69	88.5	10.4	10.0	0.796
Recurrent infections	NO	60	76.9	9.5	8.0	
	YES	18	23.1	13.0	13.0	**0.004**
Foul-smell	NO	50	64.1	9.2	8.0	
	YES	28	35.9	12.4	12.0	**0.009**
Walking problems	NO	66	84.6	10.0	9.0	
	YES	12	15.4	12.4	12.5	**0.048**
Visit/year	1	33	42.3	10.5	9.0	
	2	27	34.6	8.0	8.0	
	≥ 4	18	23.1	13.6	13.0	**0.007**
Mother works^e^	NO	39	55.7	10.4	9.0	
	YES	31	44.3	10.0	10.0	0.781
Father works^e^	NO	9	13.2	7.4	8.0	
	YES	59	86.8	10.7	10.0	0.160
Workdays lost by caregiver^e^	NO	17	23.3	7.7	7.0	
	YES	56	76.7	11.2	10.0	**0.033**

^*^Independent-samples Mann–Whitney U test for two samples, and Kruskal–Wallis 1-way ANOVA for 3 or more samples, *P* values < 0.05 are in bold

^a^LI: lamellar ichthyosis, includes 2 patients with harlequin ichthyosis

^b^CIE: congenital ichthyosiform erythroderma

^c^PPK: palmoplantar keratoderma

^d^Clinical severity score: mild-moderate 0–5, severe ≥ 6 signs and/or symptoms

^e^Totals may vary because of missing values

The mean FDLQI score for the entire sample was 10.3 (median = 10.0) (Table 1). A significantly higher burden, assessed by the total FDLQI score, was observed in family members of patients with a more severe disease score, as determined by the presence of six or more signs and symptoms (*P* = 0.003). Foul-smell (*P* = 0.009) and recurrent infections (*P* = 0.004) were the two signs that affected more severely the family QoL. Other single signs and complications significantly associated with greater family burden were: visible scales on the face (*P* = 0.039), ear deformities (*P* = 0.016), PPK (*P* = 0.041), and walking problems (*P* = 0.048). Furthermore, dark body scales (*P* = 0.094) and eclabium (*P* = 0.069) approached statistical significance. Unexpectedly, pruritus did not significantly impact the family QoL (*P* = 0.796). Finally, FDLQI scores were significantly higher for family members of patients who required ≥ 4 visits per year (*P* = 0.007).

**Fig. 2:**
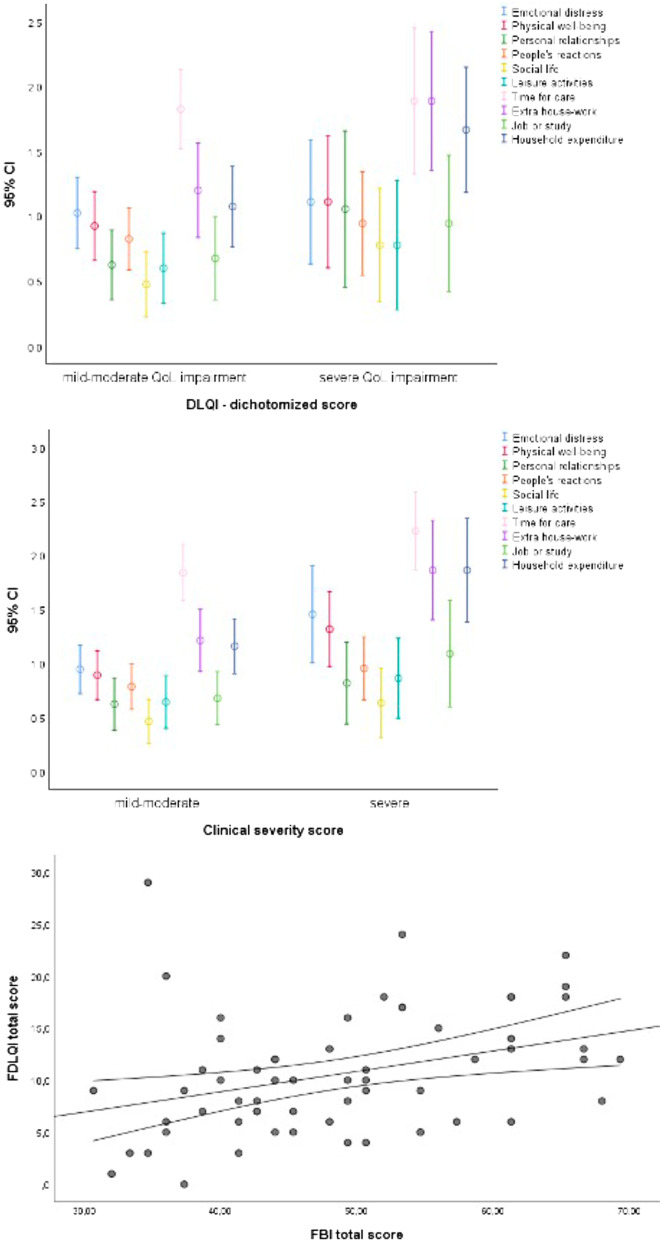
Mean and 95% confidence intervals for the scores of the 10 items of the Family Dermatology Life Quality Index (FDLQI) questionnaire stratified by the (Children) Dermatology Life Quality Index [(C)DLQI] total score: mild-moderate versus severe (**a**). Mean and 95% confidence interval, for the scores of the 10 items of the FDLQI questionnaire stratified by the clinical disease severity score: mild-moderate versus severe (**b**). Scatterplot (with fit line and 95% confidence interval) of the correlation between FDLQI and Family Burden of Ichthyosis (FBI) scores (**c**)

When considering single FDLQI items, the most affected dimensions were “time needed for care” (mean value for this item = 1.95), “extra house-work” (1.40), and “household expenditure” (1.36). Also, when stratifying for the (Children) Dermatology Life Quality Index [(C)DLQI] total score (i.e., scores < 10 versus ≥ 10) [13], as assessed in the same patient group in a recent study [12], or for disease severity score (Fig. 2a, b), these three items were the most burdensome both for mildly/moderately and severely affected QoL, as well as for the severe and less severe levels of clinical involvement. Looking at associations between single FDLQI items and disease severity, higher values for “emotional distress”, “physical well-being”, “extra house-work”, and “household expenditure” were significantly associated with the presence of ≥ 6 ichthyosis signs and/or complications (Fig. 2b). The correlation between the total (C)DLQI and total FDLQI scores was modest (Spearman’s correlation coefficient = 0.340). However, a major patient QoL impairment, as assessed by a (C)DLQI score ≥ 10, was significantly associated with higher FDLQI values for “extra house-work”, and “household expenditure” (Fig. 2a). Interestingly, the FDLQI total score showed a weak-to-moderate correlation with the FBI total score, with a Spearman’s correlation coefficient = 0.391 (Fig. 2c) [12]. In addition, the FBI total score was significantly higher in family members of LI patients as compared to CIE [12], while no differences were found in the FDLQI total score. However, when considering the single FDLQI items, greater values were obtained in LI patient caregivers as compared to CIE for “emotional distress” (mean value = 1.19 vs 0.40; *P* = 0.010), and “impact of other people’s reactions due to the relative's disease” (mean value = 0.90 vs. 0.40; *P* = 0.048).

## Discussion

The present study evaluated the impact of ARCI, as a major and highly disabling group of congenital ichthyoses, on the QoL of family members by means of a widely used dermatology-specific questionnaire, the FDLQI. Interestingly, 30 out of 78 patients, whose family members filled the questionnaire, were adults: the unusually high number of adult patients accompanied by a relative attest the need for care and support in this disease. A second relevant aspect was the high response rate, indeed, the FDLQI was not filled in 16 cases, but 12 of them corresponded to adult patients who attended the consultation alone, resulting in an actual response rate of 95.1% (78/82).

The positive association between FDLQI total score and disease severity level, as well as with frequent visits (≥ 4/year) and with single serious ARCI complications, such as recurrent infections and walking problems, could have been expected. On the other hand, clinical features which do not substantially contribute to the actual severity of disease, but do affect the patient’s body image (e.g., scales on face, dark scales, and ear deformities), were also related with a higher FDLQI total score. This finding indicates that patient’s physical aesthetic alterations may significantly impact on parents/caregivers, likely due to family concerns about emotional and social aspects of their affected relative’s life. In a recent study on the same patient group, there was no significant correlation between these signs and patient QoL, as evaluated by the (C)DLQI, while disease symptoms, in particular itch and pain, were associated with higher (C)DLQI scores [12]. It is interesting to note that itch, while being a major problem for the patients [14], does not seem to substantially affect the family members’ QoL. In addition, in the present study only a modest correlation was detected between the total (C)DLQI and FDLQI scores. Overall, these findings attest that major variations exist among factors impacting QoL of patients versus their familial caregivers. Clinicians should be aware that, even in presence of a patient with mild clinical features, family members and caregivers may suffer a considerable burden from the skin disease. Thus, our results, although showing a correlation with a more severe disease score, should not mislead to neglect psychological counselling and education warranted also in families facing milder disease. As disease manifestations affect differently the QoL of the patients and their families, a global evaluation of disease impact also on family members is an essential part of patient-reported outcomes.

No previous studies have been performed evaluating family disease burden in congenital ichthyoses using a dermatology-specific questionnaire. However, the three FDLQI items with the highest score (i.e., time for care, extra-housework, and household expenditure) in our study were similar to those reported as more affected by Gänemo and co-workers who used an atopic dermatitis-specific questionnaire (Dermatitis Family Impact Questionnaire) on parents of 15 children with different types of congenital ichthyoses [7]. They reported that the highest scores were obtained for “effect on housework”, “helping with the child treatment”, and “expenditure”. Furthermore, our findings are in keeping with the results of a French National survey aimed at characterizing the specific impact of inherited ichthyoses on patient daily life and socio-economic aspects, using an ad hoc questionnaire [15]. In that study, most patients reported additional daily housework and time spent for skin care, as well as significant out-of-pocket expenditure due to the disease [15]. Even if the National Health Systems, both in France and Italy, cover medical expenses for the care of rare diseases, housework extra costs, including house cleaning and laundry, as well as other expenses (e.g. travel costs to reference centers), pose an additional economic burden on patients and their families.

Of note, the use of FDLQI also enables comparisons with other rare skin diseases. Interestingly, the family disease burden was slightly greater in ARCI than in a severe subtype of a genetic skin fragility disease, epidermolysis bullosa (EB), recessive dystrophic EB (mean FDLQI in ARCI: 10.3 vs. 9.8 in EB) which is characterized by generalized skin blistering and unremitting wounds [16]. For dystrophic EB, the most frequently reported problems were the time spent on looking after the patient, emotional distress, physical well-being, and increased household expenditure. Thus, several variables appear common to rare and chronic skin diseases involving the entire body surface with major esthetical damage and requiring daily, time-consuming care. Finally, it is important to note that the correlation between the FDLQI and the FBI total scores was, at best, moderate in our patient group. This apparent discrepancy between specialty- and disease-specific questionnaires is not unusual, as it has been already reported, for instance, in patients with psoriasis [17]. In our particular case, the disease-specific tool addresses issues (e.g., sleep disturbance, skin smell, worry/fear of the future, acceptance/coping) that are not covered by the specialty-specific questionnaire. While the disease-specific questionnaire allows to investigate in greater detail the impact of the skin condition, it has to be kept in mind that it does not allow for comparisons with other dermatological diseases. Overall, our findings suggest that the two instruments are not superimposable, but rather complementary, and provide evidence supporting their concurrent use for a complete appreciation of the disease burden. Despite this moderate correlation, it is interesting to note that the psychological impact was the most severely affected dimension in FBI, in particular in family caregivers of LI patients [12] who present the most disfiguring clinical features. Similarly, the FDLQI identified higher emotional distress in the same group of caregivers.

## Conclusions

Our findings highlight the major burden imposed by ARCI on the QoL of family caregivers. Multiple dimensions of everyday life were heavily affected, in particular due to the time needed for care, extra housework, and household expenditure. The impact on QoL of family members was related not only to the severity of the disease and of most disabling disease complications, but also to the presence of signs mainly altering patients’ physical appearance. Altogether, our results emphasize the relevance to offer a psychological and socio-economic support to both patients and their family members, in order to guarantee an optimized global care. Finally, measuring the secondary disease impact on “the greater patient” should be part of patient-reported outcomes evaluated during clinical trials.

## Methods

### Study design and population

This cross-sectional study is part of a multicenter survey of patient-reported outcomes in ARCI, as previously described [12]. Briefly, consecutive pediatric and adult patients with a clinical diagnosis of ARCI were recruited between March 2018 and June 2019 in the Dermatological Units of five Italian reference centres for ichthyosis. Exclusion criteria were a diagnosis of a different ichthyosis form and/or refusal to give the consent to participate to the study. The study was approved by the Institutional Ethical Committee of the coordinating (Bambino Gesù Children’s Hospital) and participating centers, and conducted in accordance with the Declaration of Helsinki. Participants or their legal guardians signed the written informed consent before entering the study. All patient information were collected during the periodical follow-up clinical consultations. At the same occasion, the family caregivers completed the FDLQI questionnaire, as well as the FBI—as already reported [12]. The ARCI type was determined or verified by an experienced dermatologist based on the clinical history and features, according to the ichthyosis classification [1]. Part of the patients had also received molecular genetic diagnosis.

### Outcome measures

#### Clinical evaluation

Information was collected on patient demographics and history, family socio-economic and occupational status, frequency of disease-related consultations, consequences on school and working activities. Clinical severity was assessed using a score based on 19 signs and symptoms (i.e., ectropion, conjunctival hyperemia/corneal erosions, eclabium, ear deformity, scale size, thickness, and colour, face scaling, PPK, itch, erythroderma, fissures, ≥ 3 cutaneous infections per year, hypohidrosis, heat intolerance, foul-smell, walking difficulties, cognitive delay, and failure to thrive), as previously described [12]. Each sign/symptom was assigned a score of 1, giving a total score with range 0–19, and higher scores indicating greater clinical severity. The total disease severity score was then classified into two categories, i.e., mild-moderate for scores 0–5, and severe for scores ≥ 6. Information on molecular diagnosis and mutated gene was also collected when available.

#### Family dermatology life quality index (FDLQI)

The FDLQI is a dermatology-specific instrument, which measures the adverse impact on the health-related quality of life of the “greater patient” [11]. It consists of 10 items with possible answers on a 4-point Likert scale. The items concern the impact of a patient’s skin disease on different aspects of the family caregivers’ quality of life (emotional and physical wellbeing, relationships, dealing with the reactions of other people to a family member's disease, social life, leisure activities, burden of care, impact on job/study, housework, and expenditure). The time frame of reference for items concerns the last 1 month. The scores of individual items (0–3) are added to give a total score that ranges from 0 to 30; a higher score indicates greater impairment of QoL [11]. The validated Italian version of the FDLQI [16] was administered to the parents of all paediatric patients and also to family caregivers of adult patients who attended the consultation accompanied.

#### Family burden of ichthyosis

The FBI is a disease-specific questionnaire of which the Italian version has been produced and validated [8, 9]. It was administered to ARCI patient family members, as previously described [12], together with the FDLQI.

#### Dermatology life quality index and children’s dermatology life quality index [(C)DLQI]

The (C)DLQI are widely used dermatology-specific questionnaires to measure health-related QoL over the previous week [18, 19]. The DLQI is validated for patients ≥ 16 years old; the CDLQI can be used between ages 4 and 16 years. We used the scores of the validated Italian versions of the two questionnaires as potential predictors of burden for the “greater patient” [20]. As recommended by the authors, a cut-off of 10 defined more severe impairment of QoL.

### Statistical analysis

For the description of the study population, categorical variables were described as number and percentages, and continuous variables as mean and standard deviation. Then, for each level of the variables of interest, mean and median values of the family-centred measures were computed. Differences in the self-reported scores were tested using the Mann–Whitney U test for two samples, and the Kruskal–Wallis 1-way ANOVA for three or more samples (e.g. mutated gene). The correlation between the disease severity score and the family-reported outcomes was studied using the Spearman’s correlation coefficient. All analyses were performed with the statistical package IBM SPSS Statistics for Windows, Version 26.0.0.1 (IBM Corp., Armonk, NY, USA). Age was grouped into two categories, i.e., 1–17 and ≥ 18 years of age. We had performed subgroup analyses for patients younger than 18 years of age, and found no relevant differences between the 0–11 year age group, comprising 40 patients, and the 12–17 year group, with 8 patients. We therefore opted for the two-group subdivision to increase precision of the estimates. Finally, the number of disease-related consultations per year was grouped into three categories: once a year, once every 6 months, and at least once every 3 months.

We compared the FDLQI scores with the scores obtained from the FBI questionnaire using the intraclass correlation coefficient (ICC). The ICC is equivalent to the kappa statistic for continuous values. It has the advantage over the Pearson or Spearman correlation coefficient in that it is a true measure of agreement, combining information on both the correlation and the systematic differences between the readings. Only for this purpose, we have transformed the original FDLQI scores to a scale of 100 to obtain the same units of measurement as used by the FBI.

## Data Availability

The datasets used during the current study are available from the corresponding author on reasonable request.
